# Solar-wind–magnetosphere energy influences the interannual variability of the northern-hemispheric winter climate

**DOI:** 10.1093/nsr/nwz082

**Published:** 2019-06-25

**Authors:** Shengping He, Huijun Wang, Fei Li, Hui Li, Chi Wang

**Affiliations:** 1 Geophysical Institute, University of Bergen and Bjerknes Center for Climate Research, Bergen 5007, Norway; 2 Key Laboratory of Meteorological Disaster/Collaborative Innovation Center on Forecast and Evaluation of Meteorological Disasters, Nanjing University of Information Science and Technology, Nanjing 210044, China; 3 Climate Change Research Center, Chinese Academy of Sciences, Beijing 100029, China; 4 Nansen-Zhu International Research Centre, Institute of Atmospheric Physics, Chinese Academy of Sciences, Beijing 100029, China; 5 Norwegian Institute for Air Research, Kjeller 2007, Norway; 6 State Key Laboratory of Space Weather, National Space Science Center, Chinese Academy of Sciences, Beijing 100190, China

**Keywords:** solar wind, winter climate, interannual variability, stratosphere

## Abstract

Solar irradiance has been universally acknowledged to be dominant by quasi-decadal variability, which has been adopted frequently to investigate its effect on climate decadal variability. As one major terrestrial energy source, solar-wind energy flux into Earth's magnetosphere (E_in_) exhibits dramatic interannual variation, the effect of which on Earth's climate, however, has not drawn much attention. Based on the E_in_ estimated by 3D magnetohydrodynamic simulations, we demonstrate a novelty that the annual mean E_in_ can explain up to 25% total interannual variance of the northern-hemispheric temperature in the subsequent boreal winter. The concurrent anomalous atmospheric circulation resembles the positive phase of Arctic Oscillation/North Atlantic Oscillation. The warm anomalies in the tropic stratopause and tropopause induced by increased solar-wind–magnetosphere energy persist into the subsequent winter. Due to the dominant change in the polar vortex and mid-latitude westerly in boreal winter, a ‘top-down’ propagation of the stationary planetary wave emerges in the Northern Hemisphere and further influences the atmospheric circulation and climate.

## INTRODUCTION

As the fundamental energy source of Earth's climate, the solar irradiance can dramatically influence Earth's climate, the earliest study of which can be traced back to the early eighteenth century [1]. It is well recognized that the variability of the solar activity (e.g. sunspot number, solar radio flux at 10.7 cm) exhibits mainly the quasi-decadal variability (i.e. prominent 11-year cycle) [2–4]. In the past >100 years, there has been a growing body of evidence that the tropospheric and stratospheric climatic variables are affected by the solar activity on both global and regional scales [5–10].

In the stratosphere, the heating can be modulated by the solar cycle due to the variations in the ultraviolet absorption by ozone [11,12]. Associated with the solar maximum, the upper stratospheric zonally averaged temperature at the equator, where the solar radiative input is the largest [13], is higher compared to that at the solar minimum [14]. The increase in solar radiative input during the solar maximum can lead to 1−2°C of increasing in the zonal-mean annual temperature located below the equatorial stratopause [15,16]. Such positive temperature anomalies intensify the mean poleward meridional temperature gradient and hence lead to anomalous westerly wind at mid-latitudes of the Northern Hemisphere [17,18]. As the propagation of the planetary waves has close relation to the background zonal winds, a downward propagation of the atmospheric anomaly to mid- and high latitudes of the lower stratosphere is generally observed at the solar maximum [13,18,19]. Such a stratosphere ‘top-down’ influence provides a potential pathway through which the solar cycle influences the interdecadal variability of the tropospheric circulation and climate in the Northern Hemisphere.

It should be noted that most of the previous studies have adopted the parameters that are dominated by quasi-decadal (i.e. 11-year-cycle) variability, to investigate the solar impacts on the atmospheric circulation and climate. Such solar cycle–atmosphere/climate relationships are not a simple linear correlation on an interannual time scale, but rather a kind of decadal modulation effect of the 11-year solar cycle. For example, by comparing the solar-maximum with the solar-minimum periods, many studies have explored the different responses of the climate or investigated the different climatic impacts of other factors such as El Niño−Southern Oscillation (ENSO) [20,21], Arctic Oscillation [22,23] and North Atlantic Oscillation [24]. Actually, except for the quasi-decadal variability, higher-frequency variability also exists in solar activity [25]. It is estimated that the energy of the solar wind and cosmic rays can be increased by tens and hundreds of times during solar-maximum periods [26]. Therefore, it is very important to examine the potential influence of total energy input from the solar wind into Earth's magnetosphere (E_in_) on the interannual variability of climate, which has been rarely discussed before due to the large challenge in quantitative estimation of E_in_ [27–29]. Utilizing the E_in_ quantitatively estimated by 3D magnetohydrodynamics (see the ‘DATA AND METHODS’ section) [30], in this study, we reveal a novel influence of solar-wind–magnetosphere energy on the winter climate over the Northern Hemisphere.

## SOLAR-WIND–MAGNETOSPHERE ENERGY IS LINKED TO NORTHERN-HEMISPHERIC WINTER TEMPERATURE AND ATMOSPHERIC CIRCULATION

**Figure 1.**
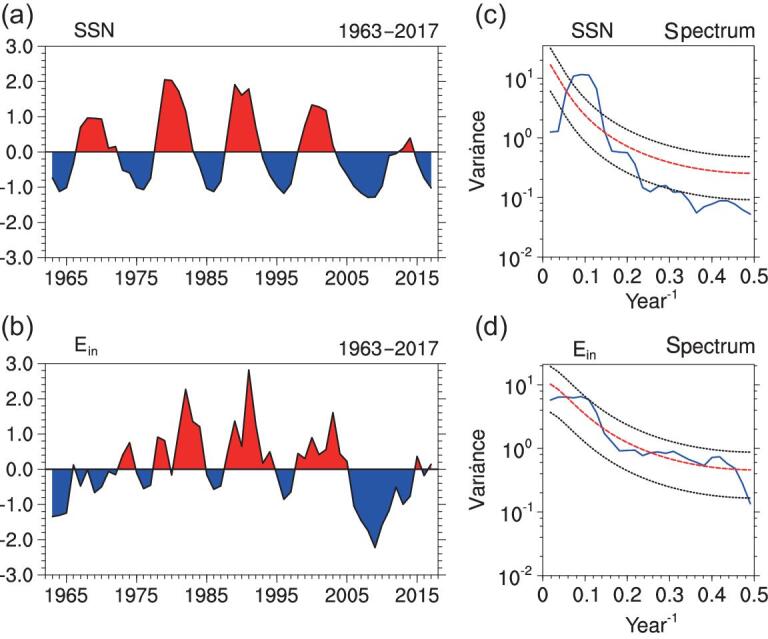
Decadal and interannual variability of solar variables. Normalized time series of annual mean (a) SSN, (b) E_in_ during 1963−2017. Spectral analysis (blue curves) for (c) SSN and (d) E_in_; the red dashed line indicates the red-noise confidence interval and the black dashed lines indicate the upper and lower confidence bounds.

The sunspot number (SSN) shows apparent low-frequency variability with alternate positive and negative phases during which no apparent high-frequency variability is observed (Fig. 1a). Even though alternate positive and negative phases are also observed in E_in_, there is obvious high-frequency variability during positive/negative phases (Fig. 1b). To illustrate clearly the time scale of the variability, we present, in Fig. 1c and d, the spectral analysis of the time series. The results indicate that the SSN is dominated by decadal variability (Fig. 1c), while the E_in_ displays both interannual and decadal variability (Fig. 1d). Therefore, it is possible to investigate the interannual relationship between the E_in_ and the atmospheric circulation and climate.

**Figure 2.**
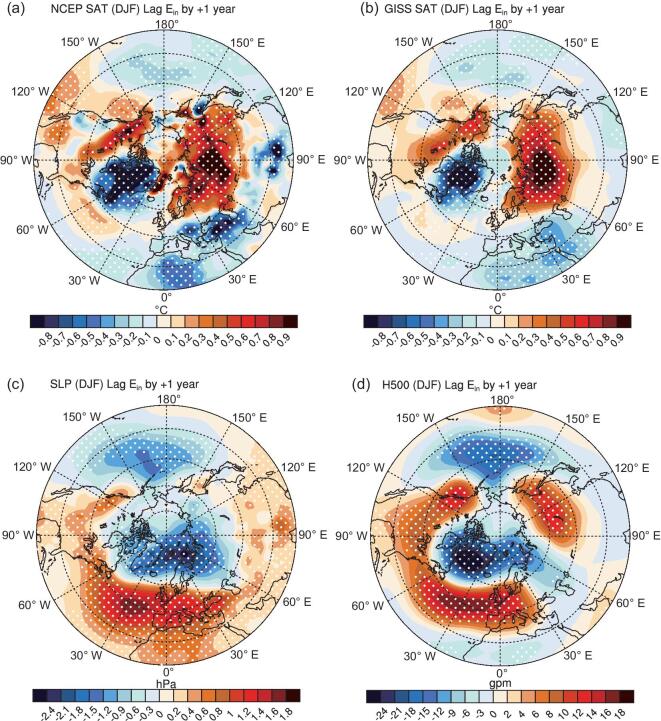
Lag relationship of boreal winter atmosphere with solar-wind energy. (a) Regression maps of surface air temperature north of 20°N during winter (December, January and February) 1964−2017 onto the normalized preceding annual mean of solar-wind energy flux into Earth's magnetosphere (E_in_) index during 1963–2016. Surface air temperature derives from NCEP/NCAR reanalysis. Dotted values are significant at the 90% confidence levels. (b) is the same as (a), but the surface air temperature derives from National Aeronautics and Space Administration, Goddard Institute for Space Studies (http://data.giss.nasa.gov/gistemp/). (c) and (d) are the same as (a), but for the sea-level pressure and 500-hPa geopotential height.

Figure 2a indicates that, during the winters following higher E_in_ in the preceding year, significantly warmer anomalies extend from northwest Europe through Siberia to northeast Asia and cross from Alaska to southwest Canada, with a maximum amplitude of up to 0.9°C over Eurasia. Meanwhile, significantly colder anomalies emerge in south Europe (south of 45°N) and East Canada to Greenland, with a maximum amplitude of up to –0.8°C. The significant lag-relationship (with maximum correlation coefficients up to 0.5) indicates the possible forcing of solar-wind–magnetosphere energy to the northern-hemispheric winter climate. The results derived from the GISTEMP Team [31] resemble those derived from NCEP/NCAR reanalysis (Fig. 2b vs. a), with a spatial correlation of 0.78. It should be noted that the results will not show much difference when the linear influence of ENSO has been removed (not shown).

Boreal winter large-scale atmospheric circulation following higher-than-normal solar-wind–magnetosphere energy is further explored. As shown in Fig. 2c, corresponding to higher-than-normal annual mean E_in_, the winters in subsequent years generally see significant positive sea-level-pressure (SLP) anomalies at mid-latitudes of the North Atlantic, with the maximum anomaly of 2.4 hPa at the central Atlantic (around 45°N). The positive SLP anomalies extend across North America through the North Atlantic eastward to the Eurasian continent. At the same time, significant negative SLP anomalies are located in the Atlantic–Arctic sector, with minimum values of −2.4 hPa centered at the Barents Sea. The spatial distribution of the winter SLP anomalies related to the higher-than-normal E_in_ in the preceding year resemble the positive-phase North Atlantic Oscillation (NAO) or Arctic Oscillation (AO) pattern. A similar NAO-/AO-like pattern is also found in the 500-hPa geopotential height anomalies (Fig. 2d). Note that there is an additional significant positive anomaly center located over the Baikal, which is missing in the SLP field, indicating the possible ‘top-down’ propagated influence of solar-wind–magnetosphere energy. Results based on the interannual variability obtained from another filtering method [32] are similar (Supplementary Fig. 1). The spatial pattern of the temperature anomalies related to the positive phase of AO/NAO (Supplementary Fig. 2a and c) resembles closely the one related to the higher-than-normal E_in_ (e.g. the spatial correlations of Fig. 2a with Supplementary Fig. 2a and b are 0.81 and 0.86, respectively). It further supports speculation that the significant interannual relationship between the solar-wind–magnetosphere energy and northern-hemispheric winter climate may be attributed to the solar impacts on the NAO-/AO-related atmospheric circulation variability (Supplementary Fig. 3).

## STRATOSPHERIC–TROPOSPHERIC DYNAMICAL ANALYSES

**Figure 3.**
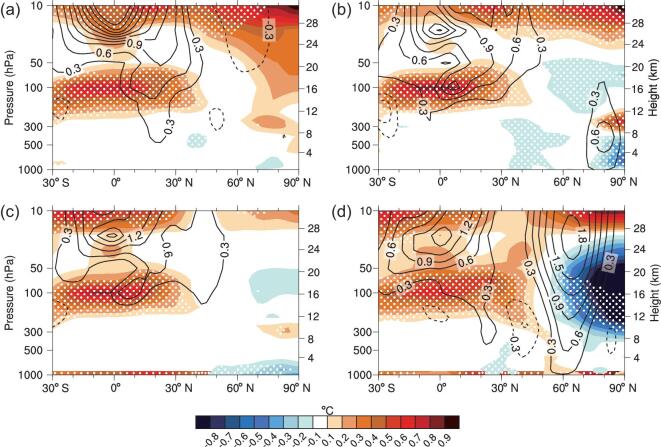
Persistence of anomalous signals in the atmosphere. Regression of zonally averaged zonal wind (contours) and air temperature (shading) in (a) March–May, (b) July–August, (c) September–November and (d) December–February during 1964–2017 onto the normalized preceding annual mean of solar-wind energy flux into Earth's magnetosphere (E_in_) index during 1963–2016. Stippled regions indicate that the temperature anomalies are significant at the 90% confidence level from a two-tailed Student's *t*-test.

Following the increase in solar-wind–magnetosphere energy, two statistically significant warming centers over the tropical region (30°S−30°N) with magnitude of up to 0.5°C emerge in the upper troposphere (200−70 hPa ≈ 12−18 km) and stratopause, persisting from boreal spring (March to April) to boreal winter (Fig. 3a−d, shading). The two warming responses might be caused by the increased solar ultraviolet irradiance and adiabatic warming as well as increased ozone heating [33,34]. In boreal winter, the higher solar-wind–magnetosphere energy intensifies dramatically the stratospheric polar vortex [19] (Supplementary Fig. 4). Due to the intensification of the polar vortex, a dominant cooling of –0.9°C emerges in the upper troposphere and lower stratosphere over the polar region (Fig. 3d, shading). Consequently, the poleward temperature gradient is further intensified in boreal winter, which induces an apparent westerly-wind anomaly at high latitudes with maximum acceleration in the speed of up to 1.8 m s^−1^ (around 60°N; Fig. 3d, contours). Note that the interannual variability of the zonal-mean air temperature, high-latitude zonal wind and polar vortex in boreal winter at lag +1 year responding to the E_in_ is in good agreement with the counterparts of the solar maximum revealed by Thiéblemont *et al.* [19]. It implies that the previously proposed pathway, on a decadal time scale, can also link the solar activity to the northern-hemispheric climate on an interannual time scale. Great potentials of impacts and applications could be explored from the solar-wind–magnetosphere energy.

Anomalous zonal background flow can alter the planetary-wave propagation from the stratosphere downward to the troposphere so as to connect the solar signals with the surface climate in boreal winter [35]. When there is higher annual mean solar-wind–magnetosphere energy, the subsequent years’ winters experience apparent anomalous E–P fluxes propagating from the upper stratosphere downward into the troposphere at latitudes north of 60°N (Supplementary Fig. 5a, vectors). The anomalous downward propagations of the winter E–P flux may be induced by the acceleration of the high-latitude westerly wind in winter (Fig. 3d, contours), which is unfavorable for the upward propagation of planetary waves [36]. The anomalous downward-propagating planetary waves lead to E–P flux divergence anomalies in the stratosphere and upper troposphere at high latitudes (Supplementary Fig. 5, shading), which further accelerates the westerly wind and strengthens the polar vortex (Supplementary Fig. 4, shading) [35,37,38]. Such a mean flow-wave interaction in the stratosphere may provide one possible way to maintain the downward propagation of the solar signals [19]. It may be noted that apparent planetary waves propagate from the lower troposphere at mid-latitudes (between 30° and 60°N) upwards to the upper troposphere (∼100 hPa) and bend equator-ward (Supplementary Fig. 5, vectors), which may be induced by the mid-latitude sea-surface temperature anomalies related to the solar-wind–magnetosphere energy [19]. Because equatorward-pointing E–P fluxes correspond to poleward meridional eddy momentum flux, the increase in solar-wind–magnetosphere energy induces eddy westerly momentum flux anomalies toward to the Arctic from the middle to upper troposphere (Supplementary Fig. 5, vectors between 300 and 100 hPa). These poleward-momentum fluxes further help to sustain the anomalous westerly at high latitudes (north 60°N), causing the downward migration of the westerly anomaly from the stratosphere downward to the troposphere so as to impact tropospheric circulation. Such a mean flow-wave interaction in the troposphere favors the influence of solar-wind–magnetosphere energy on the tropospheric circulation and climate.

**Figure 4.**
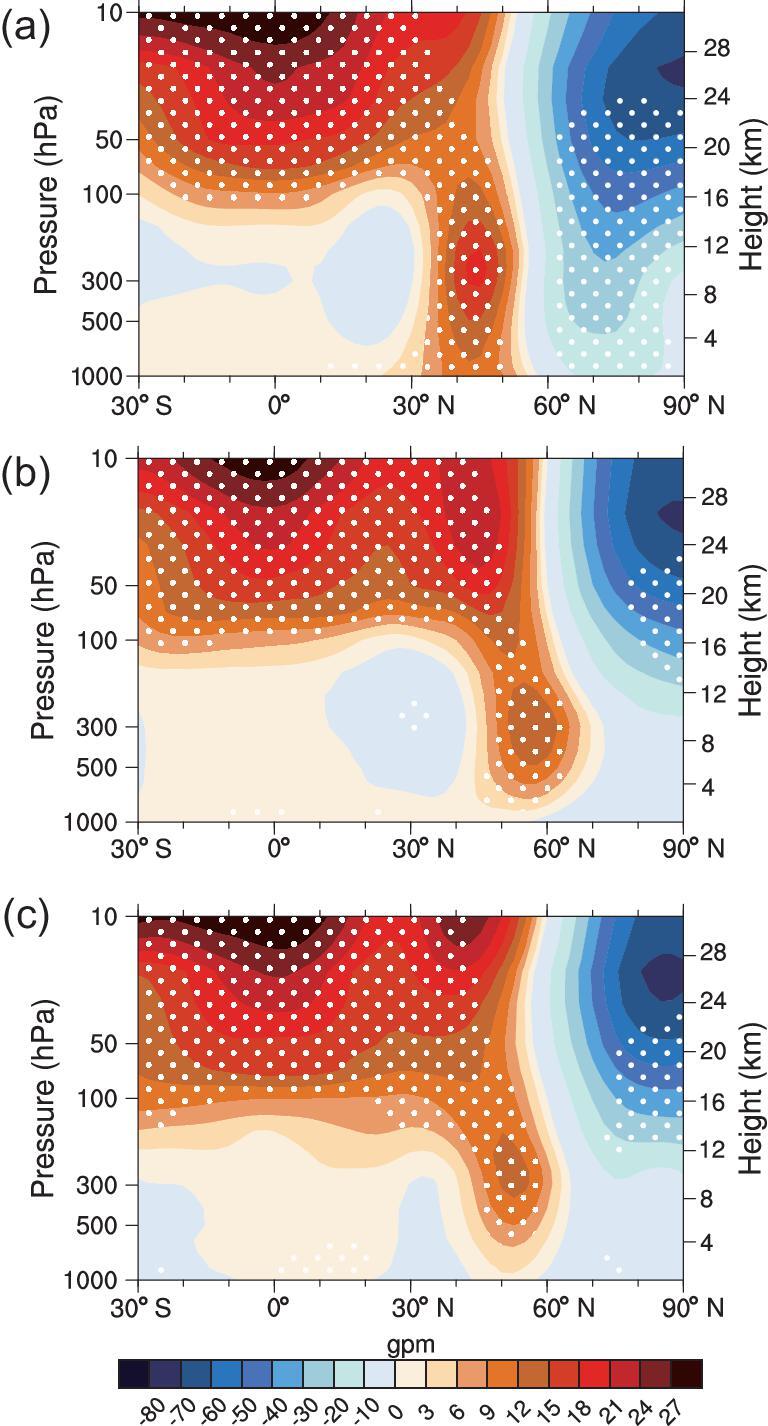
‘Top-down’ propagation of atmospheric anomalies. Vertical–horizontal cross-section for geopotential height anomalies (shading) averaged along (a) 60°W–0°, (b) 90°E–150°E and (c) 150°W–90°W during 1964–2017 winters onto the normalized preceding annual mean of solar-wind energy flux into Earth's magnetosphere (E_in_) index during 1963–2016. Stippled regions indicate that the anomalies are significant at the 90% confidence level from a two-tailed Student's *t*-test.

The downward-propagating winter atmospheric anomalies related to the solar-wind–magnetosphere energy variability is very clearly seen by inspecting the geopotential height anomalies. The geopotential height in subtropics of the North Atlantic, Eurasian continent and North America shows a significant increase in anomalies from the upper troposphere to the stratosphere during spring, summer and autumn (figures not shown). By the winter, the positive geopotential height anomalies propagate from the stratosphere downwards to the lower troposphere at mid-latitudes (Fig. 4a−c), which is most apparent over the North Atlantic (Fig. 4a). The dominant downward-propagating geopotential height anomalies at mid-latitudes in boreal winter may be related to the Brewer-Dobson circulation, which is most active in boreal winter [39,40]. Concurrently with the changes in the geopotential height, significant westerly anomalies propagate from the stratosphere downwards to the lower troposphere at high latitudes (around 60°N) (Supplementary Fig. 6a−c). Daily geopotential height anomalies also indicate that the significant positive height anomalies are located in the subtropical stratosphere before the end of November and start to propagate downwards to the troposphere at the end of November (Supplementary Fig. 7).

**Figure 5.**
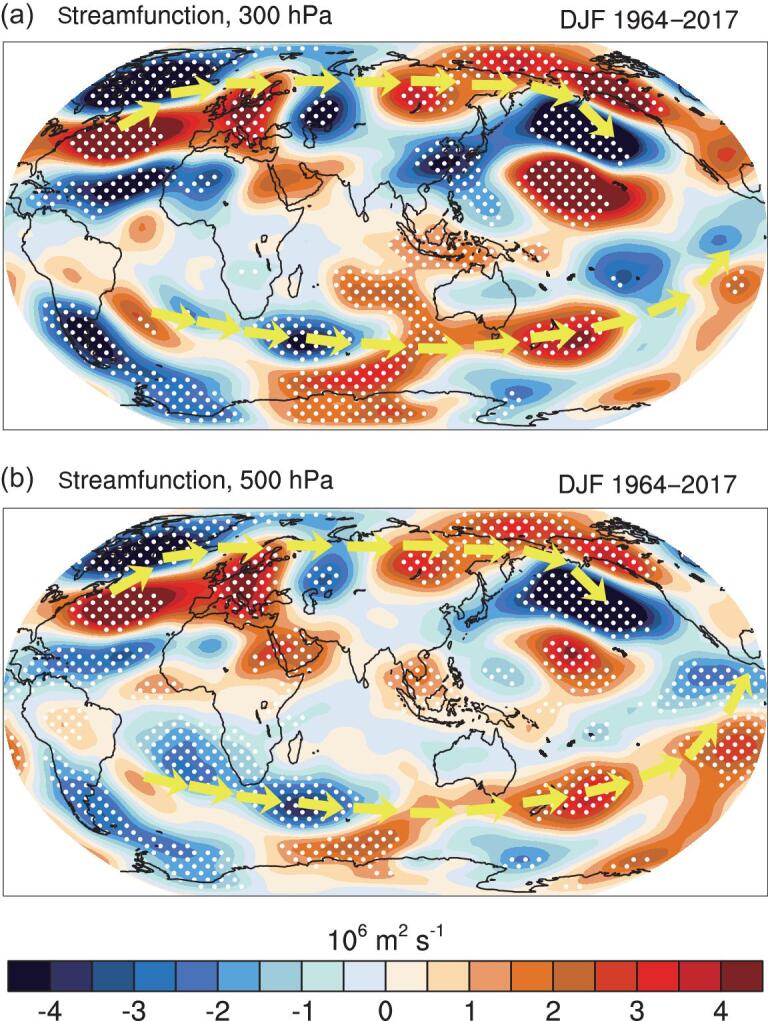
Schematic horizontal propagation of atmospheric teleconnection. Composite of stream-function anomalies at (a) 300 hPa and (b) 500 hPa between the winters (1982, 1983, 1984, 1985, 1990, 1992, 1993, 2004) following higher-than-normal annual E_in_ and those (1964, 1965, 1966, 2008, 2009, 2010, 2011, 2012) following lower-than-normal annual E_in_ in 1963–2016. Stippled values are significant at the 90% confidence levels. The yellow arrows show schematically the main wave trains emanating for the North and South Atlantic.

Consequently, two Rossby-wave-like patterns are observed in the troposphere: one propagates from the North Pacific to North America and the other propagates from the North Atlantic eastward to Eurasia (Supplementary Fig. 8a−c, vectors). The schematic is illustrated in Fig. 5. The horizontal propagation of Rossby waves can influence the blocking frequency and therefore affect the winter climate over the Northern Hemisphere [41]. The frequency of winter Ural blocking is reduced significantly by over 30% corresponding to one standard deviation of increase in the annual mean E_in_ in the preceding year (Supplementary Fig. 9a). As a result, the winter extreme cold (warm) days are significantly decreased (increased) over northern Eurasia (Supplementary Fig. 9b and c). Meanwhile, the winter blocking frequency is increased by over 25% from North America to north Europe and decreased by over 35% from north Canada to Greenland (Supplementary Fig. 9a). The significant anomalies of the blocking frequency, extremely cold days and extremely warm days in winter can explain well the anomalous winter climate over the Northern Hemisphere that is induced by the solar-wind–magnetosphere energy variability.

## CONCLUSIONS

Recent analyses of the solar/atmosphere relationship conducted by comparing two multi-decadal ocean–atmosphere chemistry–climate simulations with and without solar-forcing variability revealed a significant response of the boreal winter atmosphere at lag +1 year to the decadal variability (i.e. the 11-year solar cycle) [19]. However, the interannual relationship between the solar activity and the northern-hemispheric climate has not drawn much attention.

Based on a new index estimated by 3D magnetohydrodynamic simulations, this study reveals a novel statistically significant interannual relationship between the annual mean E_in_ and the following boreal winter climate over the Northern Hemisphere. The high variance (up to 25%, i.e. *r*^2^ = 0.25) of winter SAT over the Northern Hemisphere explained by the preceding year's solar-wind–magnetosphere energy sheds a promising improvement on climate predictions. It suggests that not only the quasi-decadal variability, but also the interannual variability of solar activity should be taken into account in climate prediction.

## DATA AND METHODS

### Energy input from the solar wind into the Earth's magnetosphere (E_in_) and atmospheric variables

The E_in_ (units: W) is estimated quantitatively using a 3D magnetohydrodynamic simulation [30]:

$$\begin{eqnarray*}
{{\rm{E}}_{{\rm{in}}}}&=& 3{\rm{.78\ \times 1}}{{\rm{0}}^{\rm{7}}}{\rm{n}}_{{\rm{SW}}}^{{\rm{0}}{\rm{.24}}}{\rm{V}}_{{\rm{SW}}}^{{\rm{1}}{\rm{.47}}}{\rm{B}}_{\rm{T}}^{{\rm{0}}{\rm{.86}}} \nonumber\\
&&\times\,\Bigg[\sin^{2.70}\left( {\frac{{\rm{\theta }}}{{\rm{2}}}} \right) + 0.25\Bigg].
\end{eqnarray*}$$

Here, *n*_SW_ and *V*_SW_ are the solar-wind number density (units: cm^−3^) and solar-wind velocity (units: km s^−1^), respectively; *B*_T_ is the transverse magnetic-field magnitude (units: nT); and *θ* is the interplanetary magnetic-field clock angle. Solar-wind data were obtained from OMNIweb (http://omniweb.gsfc.nasa.gov/) of Goddard Space Flight Center in National Aeronautics and Space Administration (NASA). Wang *et al.* [30] suggested that the E_in_ shows good performance in quantitatively estimating the energy input on a global scale.

Monthly mean and daily mean atmospheric variables were obtained from National Centers for Environmental Prediction–National Center for Atmospheric Research (NCEP/NCAR) reanalysis [42]. The monthly mean surface temperature from the Goddard Institute for Space Studies in NASA [31] was employed to support the results derived from the NCEP/NCAR reanalysis.

### Extreme events

An extremely cold/warm day is defined when a daily maximum temperature is lower (higher) than the 10^th^ (90^th^) percentile value [43]. The 10^th^ and 90^th^ percentile values are based on the daily maximum temperatures during 1981−2010. Blocking high events are defined as intervals in which daily 500-hPa geopotential height from the NCEP/NCAR reanalysis exceeds one standard deviation above the monthly mean for each grid cell over 5 consecutive days [41,44].

## Supplementary Material

nwz082_Supplemental_File

## References

[1] Herschel W . Observations tending to investigate the nature of the Sun, in order to find the causes or symptoms of its variable emission of light and heat; with remarks on the use that may possibly be drawn from solar observations. Int J Climatol 1801; 91: 85–9.

[2] Willson RC , Hudson HS. The Sun's luminosity over a complete solar cycle. Nature 1991; 351: 42–4.

[3] Camp CD , Tung KK. Surface warming by the solar cycle as revealed by the composite mean difference projection. Geophys Res Lett 2007; 34: L14703.

[4] Lean J . Solar ultraviolet irradiance variations: A review. J Geophys Res-Atmos 1987; 92: 839–68.

[5] Labitzke K , Van Loon H. Associations between the 11-year solar cycle, the QBO and the atmosphere. Part I: The troposphere and stratosphere in the northern hemisphere in winter. J Atmos Terr Phys 1988; 50: 197–206.

[6] Loon HV , Labitzke K. Association between the 11-year solar cycle, the QBO, and the atmosphere. Part II: Surface and 700 mb in the Northern Hemisphere in winter. J Clim 1988; 1: 905–20.

[7] Labitzke K , Van Loon H. The signal of the 11-year sunspot cycle in the upper troposphere-lower stratosphere. Space Sci Rev 1997; 80: 393–410.

[8] Frederick JE , Tinsley BA. The response of longwave radiation at the South Pole to electrical and magnetic variations: links to meteorological generators and the solar wind. J Atmos Sol-Terr Phy 2018; 179: 214–24.

[9] Frederick JE. An analysis of couplings between solar activity and atmospheric opacity at the South Pole. J Atmos Sol-Terr Phy 2017; 164: 97–104.

[10] Frederick JE . Solar irradiance observed at Summit, Greenland: possible links to magnetic activity on short timescales. J Atmos Sol-Terr Phy 2016; 147: 59–70.

[11] Gray LJ , Rumbold ST, Shine KP. Stratospheric temperature and radiative forcing response to 11-year solar cycle changes in irradiance and ozone. J Atmos Sci 2009; 66: 2402–17.

[12] Haigh JD . The role of stratospheric ozone in modulating the solar radiative forcing of climate. Nature 1994; 370: 544–6.

[13] Gray LJ , Scaife AA, Mitchell DM et al. A lagged response to the 11 year solar cycle in observed winter Atlantic/European weather patterns. J Geophys Res-Atmos 2013; 118: 13405–20.

[14] Mitchell D , Gray LJ, Fujiwara M et al. Signatures of naturally induced variability in the atmosphere using multiple reanalysis datasets. Q J R Meteorolog Soc 2015; 141: 2011–31.

[15] Crooks SA , Gray LJ. Characterization of the 11-year solar signal using a multiple regression analysis of the ERA-40 dataset. J Climate 2005; 18: 996–1015.

[16] Frame TH , Gray LJ. The 11-yr solar cycle in ERA-40 data: an update to 2008. J Clim 2010; 23: 2213–22.

[17] Holton JR . An Introduction to Dynamic Meteorology. New York: Academic Press, 3rd edn. 1992.

[18] Gray LJ , Beer J, Geller M et al. Solar influences on climate. Rev Geophys 2010; 48: RG4001.

[19] Thiéblemont R , Matthes K, Omrani N-E et al. Solar forcing synchronizes decadal North Atlantic climate variability. Nat Commun 2015; 6: 8268. 26369503 10.1038/ncomms9268 PMC4579852

[20] Calvo N , Marsh DR. The combined effects of ENSO and the 11 year solar cycle on the Northern Hemisphere polar stratosphere. J Geophys Res-Atmos 2011; 116: D23112.

[21] Zhou Q , Chen W, Zhou W. Solar cycle modulation of the ENSO impact on the winter climate of East Asia. J Geophys Res-Atmos 2013; 118: 5111–9.

[22] Huth R , Bochníček J, Hejda P. The 11-year solar cycle affects the intensity and annularity of the Arctic Oscillation. J Atmos Sol Terr Phys 2007; 69: 1095–109.

[23] Chen W , Zhou Q. Modulation of the Arctic Oscillation and the East Asian winter climate relationships by the 11-year solar cycle. Adv Atmos Sci 2012; 29: 217–26.

[24] Kodera K , Kuroda Y. A possible mechanism of solar modulation of the spatial structure of the North Atlantic Oscillation. J Geophys Res-Atmos 2005; 110: D02111.

[25] He S-P , Wang H-J, Gao Y et al. Influence of solar wind energy flux on the interannual variability of ENSO in the subsequent year. Atmos Ocean Sci Lett 2018; 11: 165–72.

[26] Troshichev O , Egorova L, Janzhura A et al. Influence of the disturbed solar wind on atmospheric processes in Antarctica and El-Nino Southern Oscillation (ENSO). Memorie-Societa Astronomica Italiana 2005; 76: 890–8.

[27] Akasofu S-I . Energy coupling between the solar wind and the magnetosphere. Space Sci Rev 1981; 28: 121–90.

[28] Newell PT , Sotirelis T, Liou K et al. Pairs of solar wind–magnetosphere coupling functions: combining a merging term with a viscous term works best. J Geophys Res-Space 2008; 113: A04218.

[29] Li H , Wang C, He S-P et al. Plausible modulation of solar wind energy flux input on global tropical cyclone activity. J Atmos Sol-Terr Phys 2018; 192: 104755.

[30] Wang C , Han J, Li H et al. Solar wind–magnetosphere energy coupling function fitting: results from a global MHD simulation. J Geophys Res-Space 2014; 119: 6199–212.

[31] GISTEMP Team . GISS surface temperature analysis (GISTEMP). NASA Goddard Institute for Space Studies. Land-Ocean Temperature Index, ERSSTv5, 1200 km smoothing. New York: National Aeronautics and Space Administration; 2016.

[32] Sullivan A , Luo JJ, Hirst AC et al. Robust contribution of decadal anomalies to the frequency of central-Pacific El Niño. Sci Rep 2016; 6: 38540. 27917936 10.1038/srep38540 PMC5137076

[33] Kodera K , Kuroda Y. Dynamical response to the solar cycle. J Geophys Res-Atmos 2002; 107: 4749.

[34] Cionni I , Eyring V, Lamarque JF et al. Ozone database in support of CMIP5 simulations: results and corresponding radiative forcing. Atmos Chem Phys 2011; 11: 11267–92.

[35] Hartmann DL , Wallace JM, Limpasuvan V et al. Can ozone depletion and global warming interact to produce rapid climate change? Proc Natl Acad Sci USA 2000; 97: 1412–7. 10677475 10.1073/pnas.97.4.1412 PMC26447

[36] Charney JG , Drazin PG. Propagation of planetary-scale disturbances from the lower into the upper atmosphere. J Geophys Res 1961; 66: 83–109.

[37] Andrewes DG , Holton JR, Leovy CB. Middle Atmosphere Dynamics. San Diego: Academic Press, 1987.

[38] He S , Wang H, Gao Y et al. Recent intensified impact of December Arctic Oscillation on subsequent January temperature in Eurasia and North Africa. Clim Dyn 2019; 52: 1077–94.

[39] Butchart N , Scaife AA, Bourqui M et al. Simulations of anthropogenic change in the strength of the Brewer–Dobson circulation. Clim Dyn 2006; 27: 727–41.

[40] Weber M , Dikty S, Burrows JP et al. The Brewer-Dobson circulation and total ozone from seasonal to decadal time scales. Atmos Chem Phys 2011; 11: 11221–35.

[41] Li F , Orsolini YJ, Wang H et al. Atlantic multidecadal oscillation modulates the impacts of Arctic sea ice decline. Geophys Res Lett 2018; 45: 2497–506.

[42] Kalnay E , Kanamitsu M, Kistler R et al. The NCEP/NCAR 40-year reanalysis project. Bull Am Meteorol Soc 1996; 77: 437–71.

[43] He S , Wang H. Linkage between the East Asian January temperature extremes and the preceding Arctic Oscillation. Int J Climatol 2016; 36: 1026–32.

[44] Thompson DW , Wallace JM. Regional climate impacts of the Northern Hemisphere annular mode. Science 2001; 293: 85–9. 11441178 10.1126/science.1058958

