# Supplementary material for: Solar-wind–magnetosphere energy influences the interannual variability of the northern-hemispheric winter climate
Source: Natl Sci Rev. 2019 Jun 25;7(1):141–8. doi: 10.1093/nsr/nwz082 (PMC8289023; doi:10.1093/nsr/nwz082)
Supplement: nwz082_Supplemental_File [file nwz082_supplemental_file.docx]

Supporting Information for

**Solar wind-magnetosphere energy influences the interannual variability of northern hemispheric winter climate**

HE Shengping^1,2,3^, WANG Huijun^2,3,4^, LI Fei^5,2,4^, LI Hui^6^, and WANG Chi^6^

^1^Geophysical Institute, University of Bergen and Bjerknes Center for Climate Research, Bergen 5007, Norway;

^2^Key Laboratory of Meteorological Disaster/Collaborative Innovation Center on Forecast and Evaluation of Meteorological Disasters, Nanjing University of Information Science and Technology, Nanjing 210044, China;

^3^Climate Change Research Center, Chinese Academy of Sciences, Beijing 100029, China;

^4^Nansen-Zhu International Research Centre, Institute of Atmospheric Physics, Chinese Academy of Sciences, Beijing 100029, China;

^5^Norwegian Institute for Air Research, Kjeller 2007, Norway;

^6^State Key Laboratory of Space Weather, National Space Science Center, Chinese Academy of Sciences, Beijing 100190, China

**Contents of this file**

Figures S1−S9

**Introduction**

This file provides the supplemental figures for the main manuscript.

**Figure S1 Lag relationship of boreal winter atmosphere with solar wind energy.** Same as the Figure 1 in the main text, but with another method to obtain the interannual variability. The Lanczos filter is applied to the five-month running mean anomaly, then we generate the low-pass filtered anomaly. The difference between the original five-month running mean anomaly and the low-pass filtered anomaly gives the interannual component variability.

**Figure S2 Boreal winter temperature anomalies related to the Arctic Oscillation, North Atlantic Oscillation.** (a) Regression maps of surface air temperature north of 20°N during winter (DJF) 1964−2017 onto the simultaneous normalized Arctic Oscillation index. (b) same as (a), but with regard to the NAO index. Dotted values are significant at the 90% confidence levels.

**Figure S3 Atmospheric circulation anomalies associated with solar wind energy.** Regression maps of (a) 300-hPa zonal wind (shading) and (b) 850-hPa wind north of 20°N during winter 1964−2017 onto the normalized preceding annual mean of solar wind energy flux into the Earth’s magnetosphere (E_in_) index during 1963–2016. Dotted values in (a) and shading values in (b) are significant at the 90% confidence levels. The contours in (a) indicate the climatology of zonal wind during winter 1964−2017.

**Figure S4 Winter polar vortex anomalies associated with solar wind energy.** Regression maps of (a) 50-hPa and (b) 100-hPa geopotential height anomalies north of 60°N (shading) during winter 1964−2017 onto the normalized preceding annual mean of solar wind energy flux into the Earth’s magnetosphere (E_in_) index during 1963–2016. Dotted values are significant at the 90% confidence levels. The contours in indicate the climatology in winter 1964−2017.

**Figure. S5 Stationary planetary wave propagation.** Regression of E-P flux (vectors; units: 10^7^ m^2^ s^-2^) and its divergence (shading; units: m s^-1^ day^-1^) during 1964–2017 winters onto the normalized preceding annual mean of solar wind energy flux into the Earth’s magnetosphere (E_in_) index during 1963–2016. Stippled regions indicate that the E-P flux divergence anomalies are significant at 90% confidence level from a two-tailed Student’s *t* test.

**Figure S6. Vertical structure of westerly wind.** Vertical-horizontal cross section for geopotential height anomalies (shading) averaged along (a) 60°W–0°, (b) 90°E–150°E, and (c) 150°W–90°W during 1964–2017 winters onto the normalized preceding annual mean of solar wind energy flux into the Earth’s magnetosphere (E_in_) index during 1963–2016. Stippled regions indicate that the anomalies are significant at 90% confidence level from a two-tailed Student’s *t* test.

**Figure S7** **Persistence and “top-down” propagation of atmospheric anomalies on daily time scale**. Vertical-horizontal cross section for the correlations between the daily geopotential height (from 1 March to 28 February, with 5-day low-pass filtered) during 1964/65–2016/17 and the E_in_ in 1963–2016, area-averaged in the domain over the North Atlantic 0°–50°N, 60°W–0°W. Values enclosed by rhombus are significant at the 90% confidence levels.

**Figure S8 Propagation of atmospheric teleconnection.** Regression maps of (**a**) 300-hPa and (**b**) 500-hPa streamfunction (shading, units: 10^6^ m^2^ s^-1^) and (**c**) vertical-horizontal cross section (averaged between 40°−50°N) stream-function (shading) during winters 1964−2017 onto the normalized preceding annual mean of solar wind energy flux into the Earth’s magnetosphere (E_in_) index during 1963–2016. Regions with contours indicate that the anomalies are significant at 90% confidence level from a two-tailed Student’s *t* test. Vectors display the associated wave activity flux (vector, units: m^2^ s^-2^).

**Figure S9. Extreme events anomalies associated with solar wind energy.** Regression maps of (**a**) blocking frequency which is measured as the ratio between the number of blocked days and the total number of days, (**b**) extreme cold days, and (**c**) extreme warm days during winters 1964−2017 onto the normalized preceding annual mean of solar wind energy flux into the Earth’s magnetosphere (E_in_) index during 1963–2016. Stippled regions indicate that the anomalies are significant at 90% confidence level from a two-tailed Student’s *t* test.
